# Standards of care for medical management of acromegaly in pituitary tumor centers of excellence (PTCOE)

**DOI:** 10.1007/s11102-024-01397-w

**Published:** 2024-06-04

**Authors:** Andrea Giustina, M. M. Uygur, S. Frara, A. Barkan, N. R. Biermasz, P. Chanson, P. Freda, M. Gadelha, L. Haberbosch, U. B. Kaiser, S. Lamberts, E. Laws, L. B. Nachtigall, V. Popovic, M. Reincke, A. J. van der Lely, J. A. H. Wass, S. Melmed, F. F. Casanueva

**Affiliations:** 1 Institute of Endocrine and Metabolic Sciences, San Raffaele Vita-Salute University and San Raffaele IRCCS Hospital, Via Olgettina 60, Milan, 20132 Italy; 2 Department of Endocrinology and Metabolism Disease, School of Medicine, Recep Tayyip Erdoğan University, Rize, Turkey; 3 Division of Endocrinology, University of Michigan Health System, Ann Arbor, MI USA; 4 Center for Endocrine Tumors Leiden, Leiden University Medical Center, Leiden, The Netherlands; 5 Physiologie et Physiopathologie Endocriniennes, Service d’Endocrinologie et des Maladies de la Reproduction et Centre de Référence des Maladies Rares de l’Hypophyse HYPO, Université Paris-Saclay, APHP, Hôpital Bicêtre, Le Kremlin-Bicêtre, Inserm, Paris, France; 6 Department of Medicine, Vagelos College of Physicians and Surgeons, Columbia University, New York, NY USA; 7 Instituto Estadual do Cérebro Paulo Niemeyer, Secretaria Estadual de Saúde do Rio de Janeiro, Rio de Janeiro, Brazil; 8 Department of Medicine for Endocrinology, Diabetes and Nutritional Medicine, Charité Universitätsmedizin, Berlin, Germany; 9 Harvard Medical School, Brigham and Women’s Hospital, Boston, MA USA; 10 Erasmus Medical Center, Rotterdam, The Netherlands; 11 Pituitary/Neuroendocrine Center, Brigham & Women’s Hospital, Boston, MA USA; 12 Neuroendocrine Unit, Massachusetts General Hospital, Harvard Medical School, Boston, MA USA; 13 Medical Faculty, University of Belgrade, Belgrade, Serbia; 14 Department of Medicine IV, LMU University Hospital, LMU Munich, Munich, Germany; 15 Pituitary Center Rotterdam and Endocrinology Section, Department of Internal Medicine, Erasmus University Medical Center, Rotterdam, The Netherlands; 16 Department of Endocrinology, Churchill Hospital, University of Oxford, Oxford, United Kingdom; 17 Pituitary Center, Department of Medicine, Cedars-Sinai Medical Center, Los Angeles, CA USA; 18 Division of Endocrinology, Santiago de Compostela University and Ciber OBN, Santiago, Spain

**Keywords:** Acromegaly, Octreotide, Cabergoline, Lanreotide, Pasireotide, Pegvisomant

## Abstract

**Purpose:**

A series of consensus guidelines on medical treatment of acromegaly have been produced in the last two decades. However, little information is available on their application in clinical practice. Furthermore, international standards of acromegaly care have not been published. The aim of our study was to report current standards of care for medical therapy of acromegaly, using results collected through an audit performed to validate criteria for definition of Pituitary Tumor Centers of Excellence (PTCOE).

**Methods:**

Details of medical treatment approaches to acromegaly were voluntarily provided by nine renowned international centers that participated in this audit. For the period 2018–2020, we assessed overall number of acromegaly patients under medical treatment, distribution of patients on different treatment modalities, overall biochemical control rate with medical therapy, and specific control rates for different medical treatment options.

**Results:**

Median number of total patients and median number of new patients with acromegaly managed annually in the endocrinology units of the centers were 206 and 16.3, respectively. Median percentage of acromegaly patients on medical treatment was 48.9%. Among the patients on medical treatment, first-generation somatostatin receptor ligand (SRL) monotherapy was used with a median rate of 48.7%, followed by combination therapies with a median rate of 29.3%. Cabergoline monotherapy was used in 6.9% of patients. Pegvisomant monotherapy was used in 7 centers and pasireotide monotherapy in 5 centers, with median rates of 7.9% and 6.3%, respectively.

**Conclusions:**

Current standards of care in PTCOEs include use of first-generation SRLs as the first medical option in about 50% of patients, as recommended by consensus guidelines. However, some patients are kept on this treatment despite inadequate control suggesting that cost-effectiveness, availability, patient preference, side effects, and therapeutic inertia may play a possible role also in PTCOE. Moreover, at odds with consensus guidelines, other monotherapies for acromegaly appear to have a marginal role as compared to combination therapies as extrapolated from PTCOE practice data. Presence of uncontrolled patients in each treatment category suggest that further optimization of medical therapy, as well as use of other therapeutic tools such as radiosurgery may be needed.

## Introduction

Acromegaly is a chronic, systemic disease caused primarily by a growth hormone (GH) secreting adenoma, leading to overproduction of GH and consequently insulin-like growth factor-I (IGF-I) [1]. The reported prevalence of acromegaly has increased to 70–90 cases per million and the annual incidence is consistently reported to be 5–6 cases per million [2]. The disease is associated with systemic complications which deleteriously affect quality of life (QoL) and increase morbidity and mortality [3–5]. Thus, prompt diagnosis and treatment are essential to improve patient outcomes, since long diagnostic delay [6] negatively impacts comorbidities and mortality [7–9]. Improvements in acromegaly treatment involving new medical options provide more effective multimodality treatment options, thus allowing a patient-oriented approach [10]. According to current consensus guidelines [11], the first treatment option remains surgery, which may provide immediate biochemical remission, particularly in the infrequent instance of microadenoma. As adjuvant therapy, medical treatment was recommended for patients in whom biochemical control cannot be achieved after surgery, including somatostatin receptor ligands (SRL), dopamine agonists (DA), and GH receptor antagonists (GHRA) [12]. Octreotide, recently available in both injectable and oral formulations [13] and lanreotide are recommended as first-line medical therapy [14], whereas the multireceptor-targeted SRL; pasireotide or the GHRA; pegvisomant (PEGV) are indicated as second-line medical therapy in patients resistant to octreotide or lanreotide. For selected complex patients, PEGV and pasireotide could also be used in combination therapies [12]. The DA, cabergoline is recommended only in mild acromegaly for monotherapy, or rarely in combination therapy [10, 12].

However, it is expected that with the availability of a wide variety of medical treatment options and their related efficacy, cost, and safety profiles, real-life application of guidelines may become challenging because of the lag-time between their updates. Consequently, standards of acromegaly care may not be consistent with available guidelines, also due to constraints placed by healthcare systems and insurance coverage [15].

Pituitary Tumors Centers of Excellence (PTCOE) have been proposed as an organizational model through which a dedicated multidisciplinary expert team may provide optimal care consistent with recent guidelines and improve outcomes of pituitary diseases [16, 17]. Recently, we validated criteria for definition of PTCOE based on an audit of self-reported activity by several internationally recognized tertiary pituitary centers [18]. In this survey, participating centers provided excellent diagnosis and management, consistent with previously reported theoretical criteria [16].

The aim of our study was to evaluate acromegaly medical treatment approaches and their outcomes in the centers involved in the audit [18] fulfilling the definition of PTCOE, with the goal of providing a real-life perspective for standards of medical care in acromegaly.

## Methods

The overall study design was described in detail previously [18]. Nine centers were chosen across the world as recommended by a scientific evaluating board being most likely to meet PTCOE criteria and voluntarily participated in the study [18]. Briefly, participating endocrine centers were asked to report among many other information, total number of patients, number of new patients, and overall number of acromegaly patients under medical treatment between 2018 and 2020 [18]. Information on distribution of patients on injectable or oral octreotide, lanreotide, cabergoline, PEGV, pasireotide, and combination therapies were collected from all centers. Additionally, overall biochemical control rates of medical therapy and specific control rates with each medical treatment option were provided by eight out of nine participating centers. All centers confirmed their adherence to the latest international guidelines [10] and biochemical control rates were self-reported by each center. Results were reported as total and percentage or as median (min-max). Microsoft Excel, SPSS (version 27), and GraphPad prism 10 were used for the analysis.

## Results

Median number of patients with acromegaly in follow-up for center was 206 (40–515). Median number of new patients with acromegaly managed annually in the endocrinology units of the centers was 16.3 (7–25).

Surgical resection of GH-secreting adenomas was the second most common pituitary procedure performed in neurosurgery departments of participating centers (21% of total). Remission rates of surgical procedures were 77.3% (50–100) in acromegaly patients with microadenoma and 49% (15–83.3) in those with macroadenoma [18]. Median percentage of acromegaly patients on medical treatment was 48.9% (38.5–96.9) of all patients.

All centers reported data on first generation SRLs and cabergoline monotherapies as well as on combination therapies. A median of 39 (18–161) patients per center (48.7% of medically treated patients, range 33.3–55.9%) were receiving octreotide or lanreotide as monotherapy, 7.5 (1–15) patients (6.9%, range 1.6–22.1%) were on cabergoline alone, and 32 (12–127) patients per center (29.3%, range 23.1–50.8%) were on combination therapies.

**Fig. 1:**
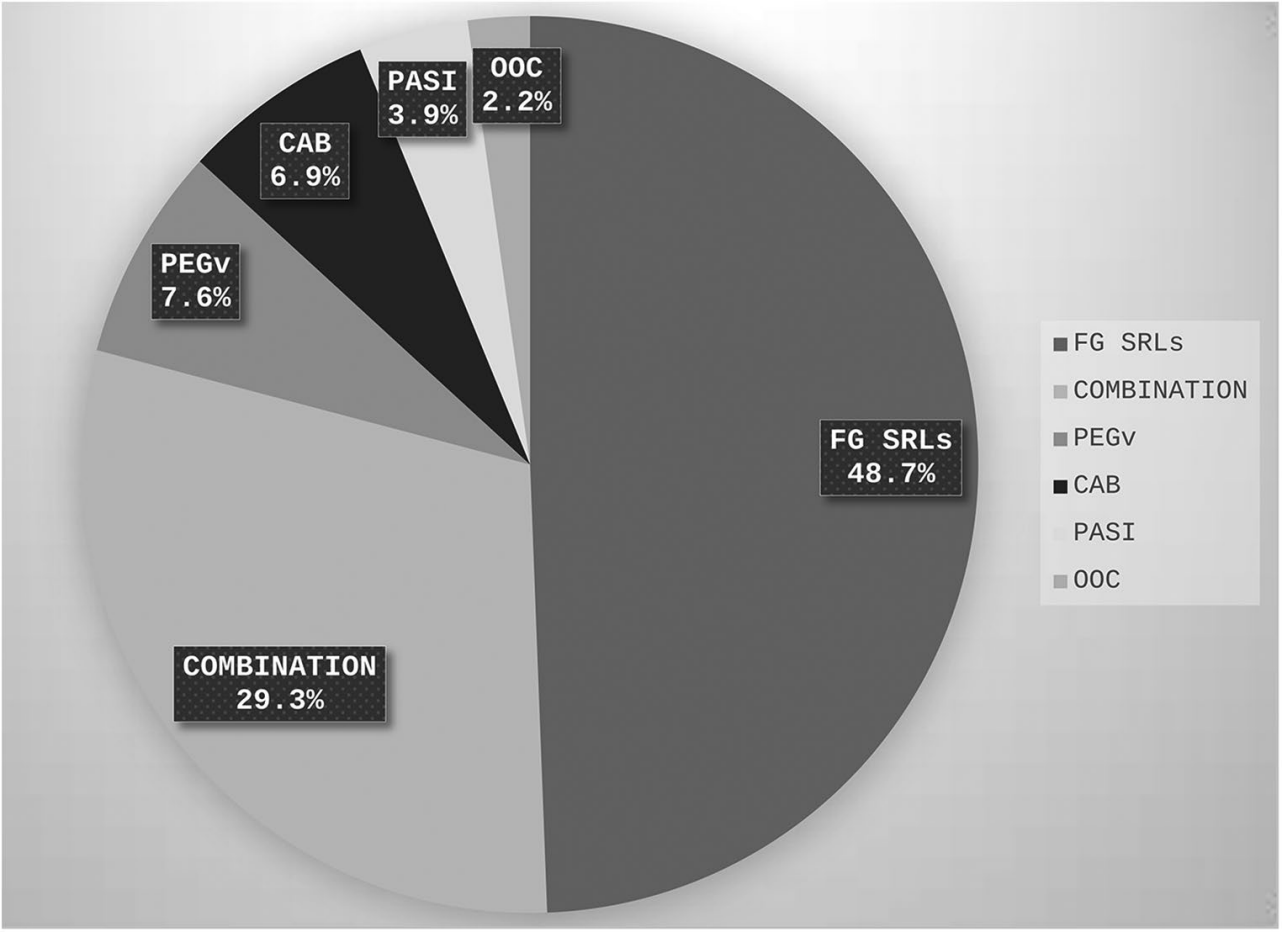
Patient percent distributions according to different medical treatment options*. *Data were provided by all 9 surveyed centers. PEGV; Pegvisomant, PASI; Pasireotide, CAB; Cabergoline, FG-SRLs; First-generation Somatostatin Receptor Ligands, OOC; oral octreotide capsule

PEGV monotherapy was used in 7 centers and pasireotide monotherapy in 5 centers (despite availability of both drugs in all centers). A median of 7.5 (0–38) patients per center (7.6%, range 0–23.2%) were on PEGV monotherapy, whereas 3 (0–28) patients per center (3.9%, range 0–22.1) were on pasireotide alone; oral octreotide therapy was used in 4 patients in a single center (2.2% of all patients under medical treatment) (Fig. 1).

**Fig. 2:**
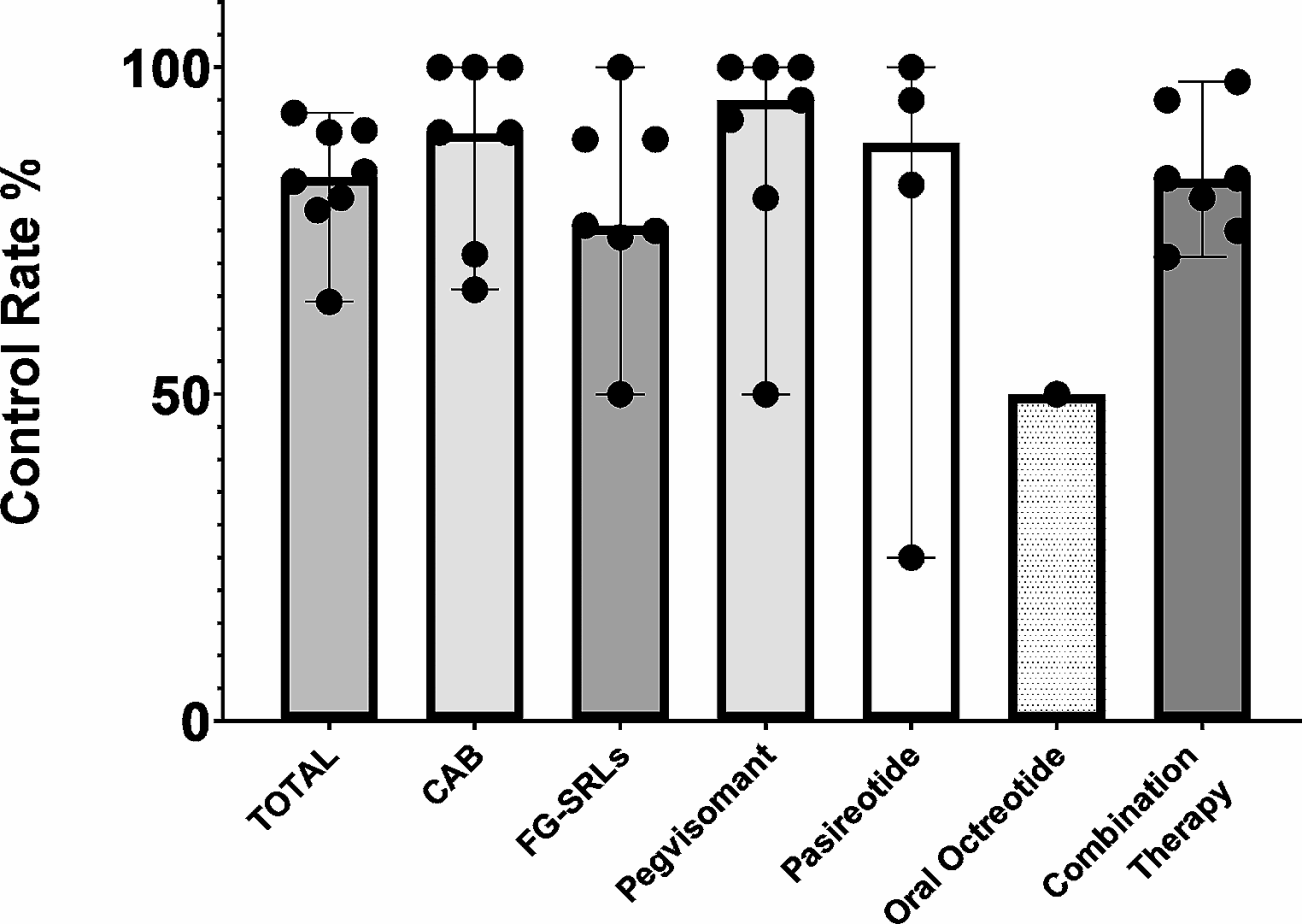
Biochemical control rates according to different medical treatment options*. Results were provided by 8 of 9 surveyed centers. Dots represent medians for each center. Bar height represents the overall median and whiskers represent the range. CAB; Cabergoline, FG-SRLs; First-generation Somatostatin Receptor Ligands. *Data for oral octreotide includes only four patients derived from single center since data was collected prior to widespread availability of this formulation

Overall, 75.8% (50–100) of patients receiving either octreotide or lanreotide as monotherapy were controlled, whereas control with PEGV or pasireotide monotherapy were achieved in 95% (50–100) and 88.5% (25–100) of patients, respectively. The control rate of cabergoline was reported to be 90% (66–100). Biochemical control with oral octreotide was 50%, but results were limited to few patients from a single center. Moreover, combination therapies provided biochemical control in 83% (71-97.8) of patients (Fig. 2).

## Discussion

This study evaluated medical treatment approaches of worldwide recognized PTCOEs for patients with GH-secreting adenomas in whom remission could not be achieved with surgery (ranging from half to one-quarter of the acromegaly population with macro- or microadenoma, respectively). The high number of patients with acromegaly reported by the centers, their expertise, and diverse geographical distribution were the elements based on which their preferred medical choices and related biochemical outcomes could represent an initial attempt to establish international standards of care in the medical management of acromegaly.

Consensus guidelines recommend octreotide and lanreotide as first-line medical treatments effective in achieving biochemical control in 40–60% of patients [19, 20]. In line with these recommendations, half of patients under medical treatment in audited centers were receiving octreotide or lanreotide monotherapy. Furthermore, while cabergoline monotherapy, used off-label in acromegaly, is recommended only for patients with mild elevations of IGF-I levels and symptoms [12], participating centers very rarely reported cabergoline monotherapy as a first option, prescribing it in < 10% of medically treated patients. Low cost and oral administration, which may allow better compliance [21], could be possible factors supporting its use as an alternative monotherapy.

According to guidelines, PEGV and pasireotide monotherapies are mainly second-line options in the treatment algorithm [22, 23], although it has been suggested that they could be used as first-line medical therapies in selected patients [24, 25]. Surprisingly, our study showed use of either PEGV or pasireotide monotherapies at a rate similar to (PEGV) or lower (pasireotide) than cabergoline monotherapy, which is known to be less effective than both drugs in achieving disease control [26]. The lower use of such second-line monotherapies (which were not used in two and four centers, respectively), compared to what would have been expected based on guidelines [10, 12, 14], may be due to their high cost and/or safety concerns. In fact, concerns for enlargement of residual adenoma tissue with PEGV [27] and hyperglycemia with pasireotide [28] have been emphasized, perhaps beyond their real impact on clinical practice patterns [29, 30].

Combination therapy is generally used due to inadequate biochemical control with monotherapy, also as a cost-effective option at least in some settings [31, 32]. Nevertheless, our results show that in real-world practice, combination therapy was the medical treatment option for one-third of patients. In fact, it appears reasonable to infer, based on our results, that in high- level centers, uncontrolled patients might have been switched directly to a combination therapy (adding either PEGV or cabergoline to octreotide or lanreotide), instead of trying monotherapy either with PEGV or pasireotide. In addition, in surveyed centers of excellence, cabergoline monotherapy appears to be a viable therapeutic option at least for some patients. In this respect, we also hypothesize that due to its modest efficacy, cabergoline monotherapy might have been switched to a combination therapy with octreotide or lanreotide for the optimization of outcomes.

Our study showed a median 83% of control rate in patients receiving medical treatment. Median control rate with octreotide or lanreotide was 75.8%, which was higher than previously reported in meta-analyses [33, 34], possibly due to inclusion of patients treated with optimal doses [35, 36]. Therefore, it is likely that these SRLs were the first option of medical treatment, as recommended by guidelines [12, 14]. Interestingly, a quarter of patients were still receiving octreotide or lanreotide monotherapy despite suboptimal biochemical control, suggesting a possible therapeutic inertia also in excellent centers [37, 38], although ongoing up titration of the dose [33, 34] as well as compliance, cost, and safety issues may also play a role [29]. Therefore, it can be inferred that there is a room for improvement in the efficacy of medical treatment. In fact, early initiation of alternative therapies in patients with risk factors who remain biochemically uncontrolled or have large tumor remnants after surgery, is one of the main objectives of PTCOEs [16–18].

Centers in the study apparently considered second-line monotherapies with PEGV or pasireotide a relatively useful alternative. In fact, despite reporting a median control rate of 95% with PEGV and 88.5% with pasireotide monotherapy (in line with the results reported in literature [39]), less than 20% of patients according to the results collected were on second-line monotherapy, whereas one-third of the overall treatment population received combination therapies.

The present study showed a median biochemical control rate achieved by combination therapy in 83% of patients, a lower rate compared to literature derived from tertiary referral centers which showed up to 95% control rates by combination therapies and restoration of normal IGF-I levels, even reaching 100% of patients in long-term follow-up [40–42], although publication bias could not be excluded. This suggests that several patients were likely switched from octreotide or lanreotide (or cabergoline) monotherapy directly to combination therapy rather than to PEGV or pasireotide monotherapy. Results may differ according to the variable impact of the safety and cost concerns in decision-making process. In fact, possible, although infrequent enlargement of residual tumor with PEGV (on which SRLs may act in a preventive way [43, 44]) and hyperglycemic effects of pasireotide (which is less notable with octreotide and lanreotide [45, 46]) might have discouraged even excellent pituitary centers from application of second-line monotherapies. In addition, combination treatments (cabergoline or PEGV added to first-generation SRLs) may be considered a cost-containing procedure that may better fit into health policies which even excellent centers may need to comply with [47].

Consensus guidelines recommend adding PEGV therapy in patients without optimal control with octreotide or lanreotide monotherapy when there are concerns about diabetes and no tumor mass [10, 12]. Further advantages of this combination can be the use of lower doses of PEGV and first-generation SRLs, leading to improved compliance by allowing decreased injection frequency [48]. The SRL and PEGV combination has been reported as a cost-effective, patient-tailored treatment option, achieving optimal biochemical control with low dose PEGV [49].

Additionally, PEGV and pasireotide combination therapy has been suggested as third-line therapy. In fact, it could provide biochemical control by individualized treatment, especially in patients with low somatostatin receptor subtype-2 expression, resistant to either first- or second-generation SRLs alone [39]. However, it is unlikely that this combination could have been frequently used in surveyed centers, as it is not yet included in guidelines. This could be considered in the future due to the possible protective effect of PEGV on the hyperglycemic action of pasireotide which in turn may offer a better tumor-directed effect than octreotide or lanreotide [50]. Cost may, however, preclude access to this treatment option. Combination of cabergoline and PEGV is unlikely to be an option for most centers, since it is not included in present guidelines and has limited additional efficacy than PEGV alone.

The main limitation of this study was the lack of detailed information on patient characteristics, indications for therapy including the role of either neuroradiological or pathological features [20], as well as specified combination treatment options, since this was a sub-study of a pilot project for PTCOE criteria validation [18]. In fact, personalized therapy which is one of the main goals of PTCOEs [51], should be offered to patients with acromegaly [49]. Despite these limitations, we believe that these results represent an interesting initial step in defining standards of medical treatment for acromegaly. In fact, it is apparent that understanding to what extent and how the translation of international guidelines into clinical practice occurs, may represent an important step and a basis for future guideline evolution. The lag-time between development of guidelines and their publication process is also a factor to be considered.

In conclusion, current standards of care of acromegaly medical treatment in PTCOEs include octreotide or lanreotide as first-line option in about 50% of patients, consistent with contemporary consensus guidelines. However, some patients have continued this treatment despite inadequate control, suggesting a possible therapeutic inertia also occuring in PCTOEs. Moreover, at odds with consensus guidelines, other monotherapies for acromegaly appear to have marginal roles compared to combination therapies in treatment algorithms of the participating centers. In fact, this initial report evaluating medical treatment practices for management of acromegaly in high-volume PTCOEs across the globe shows that one-third of acromegaly patients requiring medical therapy were on combination treatments, suggesting that in current standards of medical care, limitations of monotherapies are more frequently perceived than expected according to latest guidelines. Indeed, the cost of the drugs might have been an issue for application of guidelines into clinical practice, which may need to be carefully considered and included in future guidelines.

The clear presence of uncontrolled patients in each treatment category suggests that there could be a room for improved efficacy of medical therapy, as well as for additional therapeutic tools, such as radiosurgery [52, 53].

## Data Availability

The datasets generated during and/or analyzed during the current study are not public but are available from the corresponding author on reasonable request.

## Abbreviations

CAB: Cabergoline
DA: Dopamine agonist
GH: Growth hormone
GHRA: Growth hormone receptor antagonists
IGF-I: Insulin-like growth factor-I
PEGV: Pegvisomant
PTCOE: Pituitary Tumor Center of Excellence
SRL: somatostatin receptor ligand
